# Carotid intima-media thickness in children with end-stage renal disease on dialysis

**DOI:** 10.4103/0971-4065.62095

**Published:** 2010

**Authors:** A. Gheissari, M. Sirous, T. Hajzargarbashi, R. Kelishadi, A. Merrikhi, A. Azhir

**Affiliations:** Department of Pediatric Nephrology, Isfahan University of Medical Sciences, Isfahan, Iran; 1 Department of Radiology St Alzahra Hospital, Isfahan University of Medical Sciences, Isfahan, Iran; 2 Department of Pediatrics of St Alzahra Hospital, Isfahan University of Medical Sciences, Isfahan, Iran; 3 Department of IsfahanCardiovascular Research Center, Isfahan University of Medical Sciences, Isfahan, Iran

**Keywords:** Carotid intima-media thickness, children, end stage renal disease

## Abstract

Cardiovascular morbidity and mortality are common in end stage renal disease (ESRD) patients. There is scarce data on carotid and bulb intima-media thickness (IMT-C and IMT-B) as an early marker of atherosclerosis and related factors in children on hemodialysis (HD) and peritoneal dialysis (PD). Since we did not have enough information about our patients, this study was carried on all ESRD children (hemodialysis and peritoneal dialysis) in a referral center. Data was collected from 16 ESRD children under 18 years with seven patients on PD and nine on HD. Lab tests and biochemical parameters including serum von Willebrand factor (vWF), homocystein, apo lipoprotein A, apo lipoprotein B and quantitative CRP were measured in fasting patients just before initiating dialysis. IMT-C and IMT-B were measured by gray scale ultrasound using 7.5 MHZ probe. The mean of age was 12.76±4.5 years. The mean duration of dialysis in HD and PD patients were not significantly different; 11.88±3.25 months and 10.14±2.4 months respectively. Mean of systolic blood pressure in HD group was significantly higher than PD group, 135.55±25.54 mmHg versus 121.42±12.14 mmHg, *P*<0.05. Significant differences among all following parameters in ESRD patients, with normal laboratory values, were clarified: cholesterol, triglycerides, apo A, apo B, quantitative CRP, VWF, homocystein and IMT-C. However, we could not demonstrate any difference between IMT-B in case and control group. After adjusting for age, partial correlation showed significant correlation between IMT-C and following factors: N-PTH and serum alkaline phosphatase. Longitudinal studies with large size samples are needed to clarify the contributing factors with intima-media thickness in ESRD children.

## Introduction

Cardiovascular morbidity and mortality are common in end stage renal disease (ESRD) patients. Due to the increased survival of children on renal replacement therapy, long- term cardiovascular complications of uremia are of great concern. Among these cardiovascular complications; valvular and myocardial calcification and atherosclerosis have a great significance.

Multiple reasons for increasing the incidence of cardiovascular mortality and morbidity in ESRD patients have been considered; such as dyslipidaemia, hypertension, low grade inflammation hyperhomocysteinemia and disturbance of calcium and phosphorus homeostasis. Uremia accelerates atherosclerosis by inducing abnormal lipid metabolism and low-grade inflammation.[1] Uremia associated inflammation due to renal or systemic inflammatory disease, heart failure and dialysis dependent procedures may exacerbate atherosclerosis in chronic kidney disease(CKD) patients.[2,3]

Detecting asymptomatic patients at risk of atherosclerosis, who need more preventive modalities and medical intervention, can reduce cardiovascular diseases in children. Carotid intima-media thickness (IMT-C), an early marker of generalized atherosclerosis, can be evaluated by B-mode ultrasound.[4]

There is scarce data on carotid and bulb intima-media thickness (IMT-C and IMT-B) and related factors in children on hemodialysis (HD) and peritoneal dialysis (PD). All above mentioned markers have been proposed as risk factors for IMT-C mostly in adult ESRD patients. Since, we did not have enough information about our patients; this study was carried out on all ESRD children (on hemodialysis and peritoneal dialysis) in our referral center.

## Materials and Methods

Sixteen ESRD children under 18 years, including seven patients on PD and nine on HD from a referral center (St. Al Zahra hospital) in Isfahan, Iran, were enrolled for the study. All patients were evaluated for demographic data and routine physical examinations. All routine lab tests and biochemical parameters including serum von Willebrand factor (vWF), as a marker of endothelial dysfunction, homocystein, apo lipoprotein A (apo A), apo lipoprotein B (apo B) and quantitative CRP were measured in fasting patients, just before initiating dialysis. The mean of the three recent measurements of monthly lab tests such as calcium, phosphorus, blood urea, creatinine, n-PTH were used.

IMT-C and IMT-B were measured by gray scale ultrasound using 7.5 MHZ probe. Patients were examined in supine position with head slightly tilted to the side. Intima-media thickness were assessed at four points, 2-centimeter distal of common carotid artery (CCA) and 2-centimeter proximal of internal carotid artery (ICA) and also in two points of bulb, all in both left and right sides at the far wall. The average of values derived from CCA and ICA was interpreted as IMT-C and the average of values from both sides of bulb as IMT-B.[5,6] Data derived from the Adibi *et al*. study was used as normal range of IMT-C and IMT-B in Isfahanian children.[6]

## Results

In this study, seven male and nine females were included. The mean age was 12.76±4.5 years. The mean age of HD group was significantly greater than PD group, 16±1.89 years versus 10±4.3 years respectively. However, the mean duration of dialysis in HD and PD patients (11.88±3.25 and 10.14±2.4 months respectively) were not significantly different. Mean of systolic blood pressure but not diastolic blood pressure in HD group was significantly higher than PD group, 135.55±25.54 mmHg versus 121.42±12.14 mmHg, *P*<0.05; Table 1.

**Table 1:** Mean values of some biochemical parameters, blood pressure and also IMT-C and IMT-B in peritoneal dialysis and hemodialysis groups

	PD groupmean ±SD	HD groupmean ±SD	Significance
Calcium (mg/dl)	8.97±1.20	9.16±1.30	Not significant
Phosphorus (mg/dl)	5.08±1.43	5.98±0.95	Not significant
n-PTH (pg/ml)	267±229	2069±1427	*P = *0.008
Alkaline phosphatase (mg/dl)	615.4±372.42	1697±1229	Not significant
Cholesterol (mg/dl)	207.42±64.29	201.7±91.5	Not significant
Apo A (mg/L)	152.42±52.05	120.66±17	Not significant
Apo B (mg/L)	98.85±23.39	91.11±25.87	Not significant
CRP (mg/dl)	2.57±1.8	6.5±5.6	Not significant
VWF (IU/L)	150.36±52.4	112.83±33.04	Not significant
Homocystein (µmol/lit)	10±2.47	21.44±7.3	*P*=0.002
Ca X Ph product (mg^2^/dl^2^)	45.73±14.67	54.55±9.9	Not significant
Systolic BP (mm Hg)	121.42±12.14 mmHg	135.55±25.54 mmHg	*P*=0.023
Diastolic BP (mm Hg)	81.42±12.48 mmHg	87.77±10.63 mmHg	Not significant
IMT-C (mm)	0.467±0.77	0.505±0.147	Not significant
IMT-B (mm)	0.504±0.07	0.459±0.119	Not significant

One sample *t* test clarified significant difference among all following parameters in ESRD patients with normal laboratory values: cholesterol, triglycerides, apo A, apo B, quantitative CRP, VWF, homocystein and IMT-C. However, we could not demonstrate any difference between IMT-B in case and control group [Table 1]. IMT-C and IMT-B for normal Iranian children were derived from a recent study by Adibie's *et al*.[6]

Although mean values of phosphate binder doses and Ca×Ph product were not different in HD patients comparing to PD patients, the mean value of serum n-PTH was significantly higher in HD group. Also, the mean value of serum homocystein level as an independent risk factor for atherosclerosis was higher in HD group comparing with PD group, *P*<0.05. The mean values of more biochemical parameters of HD and PD groups are presented in Table 2.

**Table 2:** Mean values of assessed parameters in end stage renal disease patients and normal test values

Parameter	ESRD patients mean±SD	Test value upper limit	Significance *P* value
Cholesterol (mg/dl)	204.57±76.04	250	0.02
Triglyceride (mg/dl)	115.35±64.07	150	0.03
Apo A (mg/L)	134.56±38.76	190	0.0001
Apo B (mg/L)	94.5±24.32	150	0.0001
CRP (mg/dl)	4.81±4.73	3.1	0.08
VWF (IU/L)	129.89±45.03	160	0.025
Homocystein	16.43±8.09	14	0.025
(µmol/lit)			
IMT-C (mm)	0.492±0.119	0.416 (for isfahanian children)*	0.03
IMT-B (mm)	0.479±0.101	0.436 (for isfahanian children)*	>0.05

* Normal values for Iranian children were adapted from Adibie *et al*.[6]

After adjusting for age, partial correlation analysis showed significant correlation between IMT-C and n-PTH level (r = 0.85, *P = *0.04) and serum alkaline phosphatase (r = 0.86, *P = *0.02). After splitting patients into HD and PD groups a significant correlation between serum alkaline phosphatase, n-PTH and total cholesterol with IMT-C and IMT-B in HD group was shown. However, only homocystein level had significant correlation with IMT-C and IMT-B in PD groups.

Apo-A and apo-B serum levels were significantly correlated with PTH and triglyceride levels respectively, *P*<0.05.

## Discussion

Life expectancy is reduced in ESRD patients comparing with normal population. Death due to cardiovascular disease (CVD) is common among hemodialysis patients. The relative risk of CVD death in this group of patients was high as 10–30.[7–11]

Known traditional risk factors such as age, dyslipidaemia, hypertension, diabetes mellitus, smoking and sedentary lifestyle are implicated in inducing atherosclerosis in uremic patients.[12]

Also, uremia per se can increase the chance of atherosclerosis. The following non-traditional risk factors are involved in increasing the rate of atherosclerosis in ESRD patients: Oxidative stress, fat mass, impaired one- carbon metabolism, endothelial dysfunction, uremic bone disease, vascular calcification, anemia, protein- energy wasting and coagulation disorders. [13–15]

The prevalence of hyperhomocysteinemia, an independent risk factor for cardiovascular mortality and morbidity in ESRD patients, is reported as high as 85–100% in these patients. [16–18]

Lipoprotein profile, an independent risk factor of cardiovascular disease, is abnormal in CKD patients including lower HDL cholesterol, increasing the rate of apolipoprotein A1 catabolism, hypertriglyceridemia, high level of apolipoprotein B and total cholesterol but normal apo A level. [19–21] IMT-C is positively correlated with non-HDL cholesterol both in predialysis and dialysis patients.[22,23] Kumar *et al*. stated that IMT-C in ESRD children was higher than control group even before initiating dialysis.[24] A recent study on adult patients with ESRD showed a significant correlation between IMT-C and following factors: age, left ventricular mass, serum homocystein level, CRP, ESR, albumin and mean hematocrit. [25] Higher values of mean IMT-C, total homocystein fibrinogen and lipoprotein (a) in hemodialysis patients comparing with control group has been shown by Brozosko *et al*. They also revealed a positive correlation between IMT-C with the following parameters: Age, BMI, total cholesterol, LDL- cholesterol and fibrinogen but not with total homocystein or lipoprotein a. [26]

A large- sample study carried on CKD children by Litwin *et al*. represented significant correlation between IMT-C and the following factors: serum Ca×P product, the cumulative dose of calcium -based phosphate binders, and the time- averaged mean calcitriol dose. However, the cumulative phosphate binder intake, time- averaged Ca×P product and young age were shown as independent predictors of an increased IMT-C. Also, they demonstrated that IMT-C values in transplanted children and even CKD stages before evolving ESRD were lower in comparison with ESRD patients.[27]

Hakan *et al*. determined higher values of IMT-C in ESRD children comparing with control group. Also systolic, diastolic and mean arterial blood pressure were statistically higher in the ESRD group (*P*<0.05). However, multiple linear regression analysis revealed significant positive correlation only between IMT-C and left ventricular mass index (LVMI).[28]

Also, the correlation between IMT-C and duration of dialysis was clarified by Delucchi *et al*. [29] CRP has inverse correlation with creatinine clearance and has been proposed as independent predictors of intima-media thickness. [30,31] It has been suggested that endothelial injury may accelerate atherosclerosis and it is associated with many cardiovascular risk factors. The serum level of vWF, a sensitive marker of endothelial dysfunction was higher in the patients on HD with CVD than in those without.

Another study showed that vWF correlated with total cholesterol in ESRD patients. Whether vWF is causative or a by- product of the increased IMT should be evaluated in future studies. [32–35]

Lesser amounts of IMT-C in transplanted children and earlier CKD stages in comparison with ESRD patients were shown by Mieczyslaw *et al*.[27] The mean duration of dialysis in our patients was not long enough to demonstrate advanced stages of atherosclerosis. Higher amounts of parathyroid hormone and alkaline phosphatase in HD group might be a result of poorer control of hyperphosphatemia in the HD group. However, there was not a significant difference between Ca×Ph product and phosphate binder doses in both groups.

The mean age of HD group was significantly higher. It may be due to prescribing PD rather than HD for young ages especially when young children's growth and development are arrested by decreasing glomerular filtration rate (GFR) even with GFR more than 10 ml/ min. The total long-term cumulative doses of phosphate binders before getting ESRD (stage 5 CKD) are lesser in our PD patients comparing with HD group. Therefore, the increased amount of n-PTH and alkaline phosphatase in HD group is a reflection of longer duration of consuming phosphate binders and poorer control of kidney bone disease. It may partially explain the correlation between IMT-C and n-PTH in HD group.

Measurement of apolipoproteins A-I and B-100 would predict cardiovascular risk better than HDL and LDL cholesterol in clinical practice. The prevalence of dyslipidemia in our patients was high, especially in PD patients. As GFR falls, triglyceride level increases and HDL-cholesterol falls. Hypercholestrolemia as a risk factor for increased IMT-C only was shown in HD patients. Regarding the correlation between the IMT-C and hypercholesterolemia, prescribing statins in these patients might be of concern.

The conversion of homocysteine to methionine but not its transsulfuration is decreased in ESRD patients. Serum level of homocystein was higher in HD patients. However, serum homocystein played its role in increasing IMT-C only in PD patients. Isolated systolic hypertension represents a pathophysiologic process reflecting reduced arterial elasticity.

Difficulties in blood pressure control in HD children may explain the higher values of systolic blood pressure in HD group comparing with PD patients. Increasing time of each dialysis session, more fluid restriction, switching hemodialysis to peritoneal dialysis and kidney transplantation are alternative solutions to overcome this problem. However, no significant correlation was found between hypertension and IMT-C. Controlling blood pressure and also shorter duration of dialysis in comparison with other studies may reduce the role of prolonged uncontrolled hypertension in increasing IMT-C in our patients.

Quantitative CRP and vWF levels were higher in ESRD patients compared with control group, longer studies with a larger number of patients are needed to prove the role of these two factors in increasing IMT-C in children. Regarding all risk factors in inducing atherosclerosis in ESRD children, preemptive renal transplantation should be considered.
